# Memory in the wall: expanding our understanding of the roles of plant cell walls

**DOI:** 10.1111/nph.70677

**Published:** 2025-10-29

**Authors:** Hiromasa Shikata, Akira Yoshinari, Mariko Asaoka, Daisuke Takahashi

**Affiliations:** ^1^ National Institute for Basic Biology, National Institutes of Natural Sciences Okazaki 444‐8585 Japan; ^2^ Course for Basic Biology, Graduate Institute for Advanced Studies, The Graduate University for Advanced Studies (SOKENDAI) Okazaki 444‐8585 Japan; ^3^ Institute of Transformative Bio‐Molecules (WPI‐ITbM), Nagoya University Nagoya 464‐8601 Japan; ^4^ Department of Biological Sciences Faculty of Science, Kanagawa University Yokohama 221‐8686 Japan; ^5^ Graduate School of Science & Engineering Saitama University Saitama 338‐8570 Japan

**Keywords:** cell wall, lignin, mechanics, memory, plasma membrane, polysaccharide, receptor‐like kinase

## Abstract

The plant cell wall, while providing mechanical support to cells, also dynamically adjusts its composition and structure in response to cellular and environmental cues. Recent findings indicate that plants exposed to cold stress alter the composition of cell wall polysaccharides and that this altered status primes the plants to overcome future, more severe exposure. Here, we propose that the cell wall functions as a refined interface that retains information from past experiences, with such ‘memory’ being encoded as structural or compositional changes. This enables more efficient responses to future stimuli. Such a memory system may support various biological processes, in parallel with other systems such as chromatin‐based epigenetic memory. The cell wall is uniquely capable of storing higher‐order information related to the geometry of cells and tissues, mechanical cues, and environmental histories. In this context, we present emerging perspectives and representative cases of such memory stored in the plant cell wall, and we discuss plausible mechanisms through which the memory influences cellular responses.

## Introduction

While plants have achieved coordinated morphogenesis and systemic environmental responses, they have intriguingly by and large maintained the autonomy of individual cells inherited from their unicellular green algae ancestors throughout evolution. In plants, individual cells are connected to their neighbors through an extracellular structure: the cell wall. The plant cell wall is considered one of the key elements that enable plants to thrive on land, which leads to plants retaining *c*. 450 gigatons of carbon or *c*. 80% of the biomass on the planet (Bar‐On *et al*., 2018). The cell wall is a skeleton giving strength and rigidity to plant cells and shaping the whole plant body, even as it must be reconstituted after chemical and structural alterations occasioned by cell division, cell growth, and cell differentiation (Stevens, 1907). The plant cell wall with such ‘static’ and ‘plastic’ aspects has attracted the interest of researchers, and molecular structure and processing mechanisms of the cell wall have been elucidated since the 1970s (Bauer *et al*., 1973; Keegstra *et al*., 1973; Talmadge *et al*., 1973). Over the past couple of decades, the cell wall has also been recognized as a space for perceiving stimuli from external and internal environments and for processing the signals through polysaccharides and proteins located at the periphery of the cell (Wolf *et al*., 2012). The emerging concept of cell wall signaling plays a pivotal role in our understanding of how cellular processes are coordinated to adapt to fluctuating, diverse environments, and the field is now expanding greatly (Delmer *et al*., 2024). This positions the cell wall at the center of cellular processes, integrating the anatomy of individual cells – as each cell is exposed to and responds to environmental changes – and the fitness of the whole plant.

Cell walls exhibit hysteresis, where the chemical or structural states of cell walls are distinct before and after plant cells experience environmental changes, such as in ambient temperature and water availability, internal or external mechanical forces, and cell geometry linked to cell shape and spatial arrangement in tissues. Specific structural changes in the cell wall are retained in response to temperature drops and contribute to enhanced tolerance to subsequent freezing stress (Kutsuno *et al*., 2023; Takahashi *et al*., 2024). Cellulose microfibrils – fundamental cell wall elements that determine the direction of plant cell elongation – are synthesized along cortical microtubules (CMTs) (Paredez *et al*., 2006), whose orientation is aligned in individual cells in response to the internal mechanical force within the tissues (Chan *et al*., 2007). Moreover, it has been demonstrated that the heterogeneity of mechanical properties of cell walls in outer and inner tissues affects organ formation as represented by stem and seed development (Asaoka *et al*., 2021, 2023; Creff *et al*., 2023). Although compartments on the plasma membrane (PM), that is, micro‐ or nanodomains, are associated with polarized cell growth and cellular functions, heterogeneity within the wall of an individual cell at the submicron level also influences anisotropic cell growth, contributing to the complexity of epidermal cell shapes (Haas *et al*., 2020). Taken together, the hysteresis also manifests as heterogeneity in cell wall composition and structure, which likely defines the properties of individual cells, reflecting cellular information of surrounding tissues, organs, and the entire organism to be utilized for development and environmental adaptation throughout the lifespan of the plant. This suggests that plants possess ‘memory’ systems related to the cell wall.

**Fig. 1:**
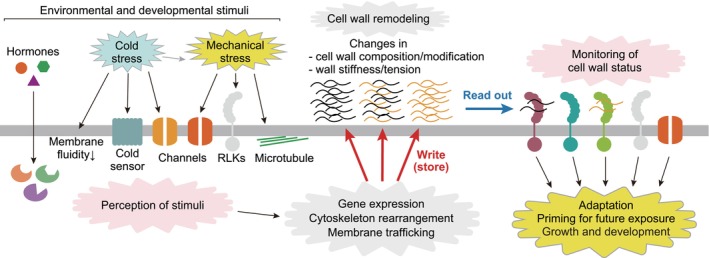
A model of cell wall memory. Environmental stimuli, such as mechanical stress and cold stress, are perceived by membrane‐localized receptors, including ion channels and receptor‐like kinases (RLKs). These receptors initiate signaling cascades that lead to changes in diverse cellular processes such as gene expression, cytoskeleton rearrangement, and membrane trafficking. Specifically, the altered expression of cell wall‐related genes and the transport of cell wall components and modifying enzymes drive cell wall remodeling, that is, modifying its composition and mechanical/chemical properties (e.g. tension and stiffness). The changes in cell wall properties are subsequently monitored by the same or additional membrane‐localized receptors, thereby triggering further intracellular signaling. These processes of stimulus‐induced remodeling (‘write/store’ of its information) and subsequent perception of the cell wall properties (‘readout’ of stored information) can be considered analogous to a memory system.

We present a concept that the plant cell wall can be considered eligible as a memory device (Fig. 1). The term ‘memory’ is often argued in animals; they form memories through synaptic plasticity, storing them in the specific patterns of their neural networks. While animals have developed nervous systems to sense environmental stimuli and store information, plants adapt to fluctuating environments by developing cell‐autonomous and systemic mechanisms without relying on a centralized system. The memory in plants is observed in a wide range of biological phenomena, and three types of categories are proposed based on the duration of memory retention: sensory memory (0.1–3.0 s), short‐term memory (minutes), and long‐term memory (up to a lifespan) (Trewavas, 2003; Volkov, 2018). Memory in the cell wall might be categorized into short‐term and long‐term memories. Memory formation machineries in plants are explained by persistent level changes in signaling metabolites, such as abscisic acid, transcription factors, and chromatin states as a consequence of stress exposure, primarily leading to alterations in the transcriptome (Crisp *et al*., 2016). Another aspect of memories is that they can be erased and reset (Iwasaki & Paszkowski, 2014; Crisp *et al*., 2016; Hilker & Schmülling, 2019). Looking at the cell wall, the composition and structure are spatio‐temporally controlled not only at the single‐cell and subcellular levels but also at the multicellular level, and the status, such as accumulation and modification, is changeable and sometimes reversible. For instance, synthesized, deposited wall compounds, for example, cellulose, hemicellulose, and pectin, are removed by degradation, and modifications such as pectin de‐methylesterification, xylan acetylation, cleavage/re‐joining of xyloglucan, and sugar compositions of polysaccharide chains are flexible. The chemical and physical record can be retrieved as information stored in the cell wall through its sensing mechanisms (Fig. 1).

Here, we define ‘memory’ in the cell wall as a process in which exposure to environmental or intrinsic stimuli causes structural or compositional changes in the cell wall, which may later be utilized to enable more efficient responses to subsequent stimuli. This article aims to explore this idea from four perspectives: mechanical interactions during development; cold‐induced structural changes in the cell wall; spatial organization through membrane–wall interactions; and mechanisms by which plants use the memory. Together, these views provide a conceptual framework for understanding how the cell wall integrates past stimuli into future cellular behavior.

## Memory of mechanics

Plant cell walls surpass many other cellular structures in complexity. This complexity arises from the diverse array of their components, including polysaccharides, proteins, and phenylpropanoids such as lignin. The composition of cell walls varies considerably among plant species, tissues, and developmental stages (Burke *et al*., 1974; Knox, 2008; Rancour *et al*., 2012). Broadly, cell walls are classified into two main types: primary cell walls (PCWs) and secondary cell walls (SCWs). PCWs consist predominantly of polysaccharides, whereas SCWs contain both polysaccharides and the phenolic polymer lignin. The PCW, the first wall formed in all plant cells, is composed of a fibrous network of cellulose microfibrils embedded in a matrix of pectins, hemicelluloses, and proteins (O'Neill & York, 2003). I contrast to the presence of PCWs in all streptophytes, SCWs first appeared in tracheophytes. SCWs are produced in specialized cells requiring increased mechanical strength, such as sclerenchyma, tracheids, and xylem vessels (Meents *et al*., 2018; Bauer *et al*., 2024). This explains the prominence of SCWs in woody plants, which contain up to 60% cellulose (McNeil *et al*., 1984), in some cases 40% lignin (Campbell & Sederoff, 1996), or 40% hemicellulose (Scheller & Ulvskov, 2010). Glycoproteins and cell wall‐associated enzymes are other key players in the regulation of cell wall architecture and dynamics (Cosgrove, 2005; Showalter & Basu, 2016).

The cell wall forms the basis of plant mechanics by conferring strength to the plant cell, and cell walls reinforce the structure of multicellular plants. Importantly, the cell wall reflects the history of its mechanical state, including the past generation of stress from turgor pressure, cell growth, or external stimuli. In this sense, the growing cell wall can itself be regarded as a record of past and ongoing mechanical cues. This mechanical guidance of directional cell growth (i.e. cellulose synthesis) not only contributes to the physical strength of the cell but also sustains the plant body plan across multiple scales (Trinh *et al*., 2021). Because CMTs align with the direction of maximal cortical tension, they guide cellulose synthesis and reinforce the wall accordingly (Chan *et al*., 2007; Hamant *et al*., 2008). During root growth, for instance, cellulose bundles reorient from transverse to longitudinal directions, thereby restricting expansion along the axis of cellulose alignment (Anderson *et al*., 2010). These observations suggest that persistent and substantial mechanical inputs can be embedded as an anisotropic ‘mechanical memory’, which may constrain growth at a specific face of the cell wall.

**Fig. 2:**
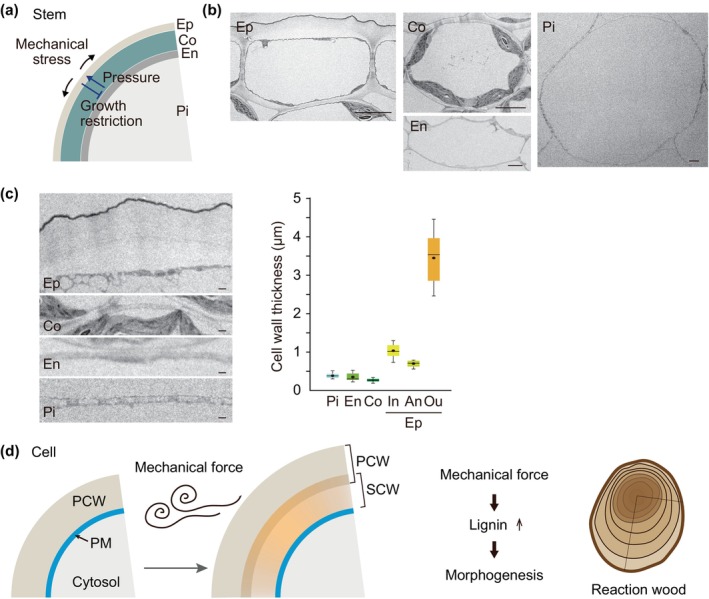
Cell wall mechanics and morphology in plant cells and tissues. (a) A schematic illustration of the mechanical conflict in a cross‐section of a cylindrical stem organ. (b, c) Transverse sections of the wild‐type stem of Arabidopsis captured by transmission electron microscopy (TEM). The images were obtained by re‐analyzing an unpublished subset of the dataset originally reported in Asaoka *et al*. (2023). Inflorescence stem portions were collected 2–3 cm above the base of the main stem at 40 d after sowing. Representative images of epidermal, cortical, endodermal, and pith cells in the inflorescence stem are shown in (b). Bars, 5 μm. Representative magnified images of the cell wall in these cells are shown in the left panel of (c). The image of the epidermal cell shows the cell wall on the stem surface. Bars, 1 μm. The quantified cell wall thickness in each cell type is displayed in the right panel of (c). Boxplots show quartiles (box limits), medians (horizontal lines), and means (dots). Whiskers indicate the total range (minimum and maximum values). *n* = 16, except *n* = 8 in endodermal cell wall. An, anticlinal wall; Co, cortical cell; En, endodermal cell; Ep, epidermal cell; In, inner periclinal wall; Ou, outer periclinal wall; Pi, pith. (d) A schematic diagram of secondary cell wall (SCW) development in plant cells as a response to mechanical forces. PCW, primary cell wall; PM, plasma membrane. For example, mechanical forces enhance lignin deposition, thereby influencing morphogenesis observed in reaction woods.

At the tissue and organ level, the spatial organization of the wall reflects mechanical experiences that guide morphogenesis. Epidermal cells, positioned in the outermost layer of an organ, provide a representative case where mechanical inputs may be recorded as ‘mechanical memory’, since they experience persistent tension during organogenesis (Fig. 2). In cylindrical organs (e.g. stem), the outer cell wall grows thick and multilayered compared to the inner one (Fig. 2), reflecting the local level of mechanical stress experienced by the epidermal cell wall (Hejnowicz, 2000; Asaoka, 2024). The tight and unbroken organization of the epidermis, the load‐bearing layer, is believed to restrict the growth of inner tissues (Kutschera & Niklas, 2007; Galletti *et al*., 2016; Verger *et al*., 2018; Asaoka *et al*., 2021). Arabidopsis pavement cells provide another example. Their jigsaw‐piece shapes with lobes and necks are thought to be favorable for increasing their size while bearing excessive mechanical stress (Sapala *et al*., 2018). Morphogenesis is controlled by a complex mechanism integrating both mechanical stress and chemical inputs like hormones (van Spoordonk *et al*., 2023), and the correlation between the heterogeneities in the cell wall and lobe initiation suggests that anisotropic mechanical forces may drive this morphogenetic process. Pectin de‐methylesterification, which is crucial for the regulation of cell expansion and morphogenesis, occurs asymmetrically in pavement cells; highly methylesterified and de‐methylesterified pectins accumulate in the lobe and indentation regions, respectively (Majda *et al*., 2017; Altartouri *et al*., 2019; Haas *et al*., 2020; Lin *et al*., 2022). Such heterogeneity in the pavement cell wall is observed before lobe formation, and this spatial information itself influences the intracellular signaling for pavement cell morphogenesis, in addition to its mechanical effect (Altartouri *et al*., 2019; Lin *et al*., 2022). While it remains unknown what initiates the heterogeneity, Tang *et al*. (2022) reported that mechanical stress activates a small GTPase Rho‐of‐Plants 6 (ROP6) through a cell wall integrity sensor, which controls lobe formation. This implies the association between mechanical inputs and the cell wall heterogeneity (see also in the ‘Receptor‐like kinases monitoring pectin status’ section).

At a broader scale, recent research focusing on the spatio‐temporal dynamics has shown that the hidden, local mechanical status of the cell wall is important for development. In the shoot apical meristem, for example, the slow‐growing area at the shoot tip is substantially strain‐stiffened compared to the surrounding fast‐growing tissue, influencing cell differentiation (Kierzkowski *et al*., 2012). Moreover, during seed development, two distinct mechanical responses occur in two different layers of the seed coat and at separate stages, which control both the seed growth rate and seed growth anisotropy, thereby determining both seed size and shape (Bauer *et al*., 2024). These examples highlight how mechanical inputs can be embedded in cell wall states that influence subsequent developmental outcomes.

These diverse examples, from cellulose reorientation during root growth to the thickened outer walls of epidermal cells and the strain‐stiffened shoot apex, illustrate that mechanical cues can leave structural imprints at multiple scales. However, not all of these imprints should be interpreted as memory. However, a key caveat is that it remains challenging to distinguish transient from persistent mechanical inputs. Transient inputs may cause temporary changes in cellulose orientation or wall elasticity, whereas only persistent or repeated inputs are generally retained as structural memory. Importantly, while transient inputs alone should not be regarded as memory, they may nevertheless trigger stable wall modifications that persist beyond the initial stimulus. In such cases, the consequences of a transient event could still be memorized. This limitation must be recognized when applying the memory concept to mechanical stimuli. Another important distinction is between direct and sensing‐mediated consequences. Some wall modifications directly constrain morphogenesis (e.g. cellulose reorientation limiting anisotropic growth), while others are perceived through cell wall integrity sensors to activate downstream signaling. Both processes may originate from mechanical inputs, but they represent distinct pathways by which experience can be memorized, and future studies will need to distinguish between these pathways. Linking back to our definition of cell wall memory as ‘persistent structural changes that modulate subsequent responses’, only long‐lasting modifications induced by mechanical cues with consequences for future developmental responses should be considered true memory.

Finally, SCW formation provides longer‐term examples of mechanical memory. Stress‐induced lignin polymers (‘stress lignin’) contrast with developmentally deposited lignin (Cesarino, 2019). Reaction wood in gymnosperms and tension wood in angiosperms are well‐documented examples of wall modifications that respond to mechanical cues (Takata *et al*., 2021) (Fig. 2). Because cell walls are extremely stable and persist after cell death, they can continue to influence neighboring living cells structurally or mechanically, functioning as a form of legacy memory. Although the molecular mechanisms of SCW signaling remain poorly understood, these cases illustrate how stress‐related regulation contributes to wall‐based mechanical memory. Functionally, PCWs provide the flexibility and plasticity required for cell expansion and morphogenesis, whereas SCWs confer rigidity and long‐term stability. This contrast suggests that different types of mechanical memory may be embedded in distinct wall systems.

## Memory of temperature changes

The polysaccharides and lignins in the cell wall are constructed from monosaccharides and monolignols, respectively. Plants synthesize > 10 monosaccharides, including glucose, galactose, and l‐arabinose, which contribute to the diversity of polysaccharide structures. Lignins, on the other hand, are primarily composed of three monolignol types: syringyl lignin (S‐lignin), guaiacyl lignin (G‐lignin), and p‐hydroxyphenyl lignin (H‐lignin) (Vanholme *et al*., 2019). Information on how these building blocks compose the cell wall provides us with valuable insights, as it allows us to infer the structural characteristics of the cell wall.

Numerous studies have demonstrated that cell wall composition is dynamic and responsive to environmental circumstances. Abiotic stresses such as heat, salinity, drought, cold, and freezing can alter the structure and composition of the cell wall (Le Gall *et al*., 2015; Tenhaken, 2015). Among these, temperature changes have been shown to trigger particularly pronounced and persistent modifications. When exposed to non‐freezing low temperatures, many plants initiate a physiological process called cold acclimation, which enhances their tolerance to subsequent freezing events. This process involves large‐scale transcriptional and metabolic reprogramming, including the accumulation of soluble sugars, late‐embryogenesis abundant proteins, and antioxidants, as well as adjustments in hormone signaling and membrane composition (Thomashow, 1999). Although much attention has been paid to cytoplasmic responses, several studies have revealed that the cell wall also undergoes significant remodeling during cold acclimation.

In particular, polysaccharide composition and cross‐linking within the wall are dynamically adjusted in response to prolonged low temperature, contributing to enhanced structural resilience. For instance, above‐freezing low temperature increases the levels of pectin, hemicellulose, lignin, and arabinogalactan proteins (AGPs) as well as simple sugars (e.g. glucose, fructose, and sucrose), which latter increase by at least 1.5‐fold after 7 d of treatment in Arabidopsis (Kutsuno *et al*., 2023). In particular, cold‐induced remodeling of xyloglucan, a hemicellulosic polysaccharide, appears to influence the capacity for cold acclimation, and the accumulation of β‐1,4‐galactan side chains on rhamnogalacturonan‐I (RG‐I) domains in pectin contributes to enhanced freezing tolerance during cold acclimation in Arabidopsis. Sub‐zero freezing temperatures, during which ice crystals form in the apoplast (extracellular space), prompt plants to reinforce their cell walls and reduce pore size, thereby limiting ice‐induced damage (Ashworth & Abeles, 1984; Panter *et al*., 2020). Previous studies have shown that boron cross‐linking between rhamnogalacturonan‐II (RG‐II) domains in pectin significantly enhances basal freezing tolerance (Panter *et al*., 2019). In *Allium fistulosum* (Japanese bunching onion), cold acclimation induces calcium‐mediated cross‐linking of homogalacturonan, leading to a reduction in apoplastic pore size and potentially limiting extracellular ice propagation. Similarly, in Arabidopsis, reduced pectin methylesterification enhances freezing tolerance, likely by promoting calcium‐mediated cross‐linking within the wall matrix (Chen *et al*., 2018; Liu *et al.*, 2022). This change likely contributes to decreased pore size and increased cell wall strength during cold acclimation. In woody plants, calcium cross‐linking between pectin‐like polysaccharides accumulates in xylem parenchyma cells during winter. These structures appear to be essential for maintaining deep supercooling in tissues by physically blocking the propagation of extracellular ice into sensitive regions, thereby functioning as a structural barrier (Wisniewski & Davis, 1989).

Compositional changes in lignin have also been reported to occur in response to various stimuli, including low temperature and wounding (Cesarino, 2019; Han *et al*., 2022). In the shoots of 3‐month‐old poplar (*Populus tremula* × *P. tremuloides* L. cv Muhs1) seedlings, lignin content increases when subjected to low temperatures (10°C) (Hausman *et al*., 2000). Since lignin is rarely degraded once deposited, the lignin accumulated under low‐temperature conditions is likely retained during subsequent growth. However, this response is tissue‐specific and not observed in cultured cells (Hausman *et al*., 2000). This suggests that low temperature‐induced lignin deposition is regulated at both cellular and tissue levels, warranting further investigation into the significance of both the composition and localization.

**Fig. 3:**
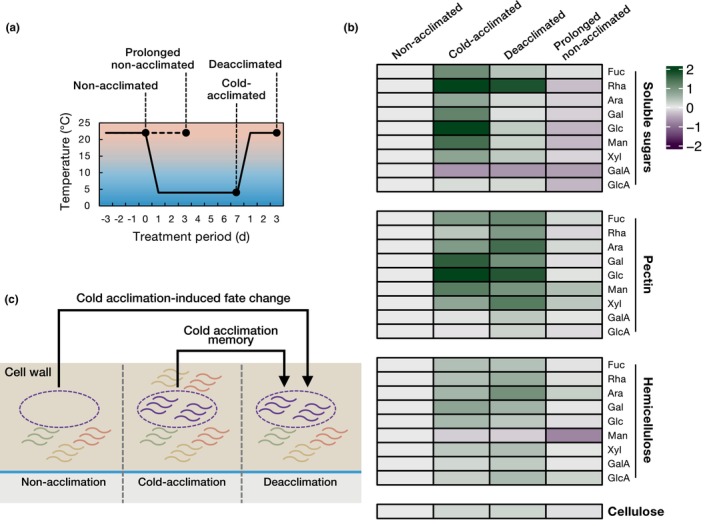
Changes in soluble sugars and cell wall constituent sugars during cold acclimation and deacclimation. (a) A diagram of temperature changes for cold acclimation/deacclimation. Arabidopsis plants were grown at 22°C (Non‐acclimated), subjected to cold acclimation at 4°C for 7 d (Cold‐acclimated), and then returned to 22°C for 3 d (Deacclimated). Non‐acclimated plants were continuously grown at 22°C for 3 d and used as growth controls for deacclimated plants (Prolonged non‐acclimated). (b) A heatmap displaying the changes in the sugar contents during cold acclimation/deacclimation. The amounts (μg mg^−1^ fresh weight) of each monosaccharide in the hydrolysis products of the soluble sugar, pectin, hemicellulose, and cellulose fractions were obtained from Kutsuno *et al*. (2023). The Log_2_‐transformed fold changes relative to non‐acclimated plants were represented by the indicated color keys. Values represent means of *n* = 3. Fuc, l‐fucose; Rha, l‐rhamnose; Ara, l‐arabinose; Gal, galactose; Glc, glucose; Man, mannose; Xyl, xylose; GalA, galacturonic acid; GlcA, glucuronic acid. (c) A schematic diagram of cold acclimation‐induced changes in cell wall sugar dynamics. Once exposed to cold acclimation, specific cell wall components are retained even after deacclimation rather than being degraded. This suggests that their fate differs from those of non‐acclimated cells, and that the experience of cold acclimation is memorized in the cell wall.

Once environmental stress is alleviated, such as after a cold period, plants must quickly resume normal growth and development to optimize energy use. After cold acclimation, freezing tolerance is rapidly lost upon warming, and growth is resumed – a process known as deacclimation (Oono *et al*., 2006; Vyse *et al*., 2019; Kutsuno *et al*., 2023). However, while many cellular processes revert to their pre‐cold acclimation state, the cell wall composition does not fully return to its original state. For example, in *Arabidopsis thaliana*, cold acclimation significantly induces the accumulation of soluble monosaccharides, which largely return or tend to return to non‐acclimated levels upon deacclimation (Fig. 3). However, the amounts of constituent sugars derived from pectin and hemicellulose do not return to non‐acclimated levels (e.g. xylose in hemicellulose) and some even increase (e.g. l‐arabinose in pectin) after deacclimation (Fig. 3). Furthermore, a clear difference in the amount of each cell wall constituent sugar is observed when comparing plants that were continuously grown at room temperature without cold acclimation (prolonged non‐acclimated plants) to those that underwent cold acclimation followed by deacclimation (Fig. 3). This suggests the possibility that phenomena distinct from simple growth or developmental progression occur in the cell wall. In other words, partial structural changes in the cell wall induced by cold acclimation may be retained even after the low‐temperature conditions are removed (Kutsuno *et al*., 2023). Such retained modifications may provide structural cues that could influence the future fate of each cell by altering its physical properties and developmental potential (Fig. 3). Moreover, in oilseed rape, the activity of pectin methylesterase (PME), an enzyme that modifies methyl groups in the main chain of pectin (homogalacturonan) to enhance calcium cross‐linking, increases during cold acclimation and remains elevated even during deacclimation (Solecka *et al*., 2008). While the concept of memory is often linked to transcriptional changes through epigenetic regulation, as seen in vernalization (Sung & Amasino, 2004; Berry & Dean, 2015; Kim *et al*., 2015), this form of memory is fundamentally different from structural modifications that are retained in the cell wall. Epigenetic memory entails stable changes in gene expression, while structural memory in the cell wall refers to enduring alterations in its physical properties or architecture that can influence later developmental outcomes. For example, calcium cross‐linking of pectin domains, which remains after cold exposure, could change tissue rigidity and cell expansion potential, which could function as a structural memory embedded in the extracellular space.

The biological significance of this ‘structural memory’ in the cell wall, particularly regarding temperature history, remains largely unknown. Although it is still uncertain whether and how these specific cell wall modifications are read out by the cell, we hypothesize that such persistent changes act as a form of ‘priming’ or ‘pre‐setting’ of the cellular signaling machinery. For instance, the altered pectin or hemicellulose composition observed during cold acclimation could prime specific PM‐localized cell wall integrity (CWI) sensors, as discussed in the ‘Role of RLKs and calcium signaling during cold acclimation’ section, by modifying their ligand binding affinity or activation threshold. This sensing mechanism may lead to a more rapid and robust activation of downstream signaling cascades (e.g. MAPK activation and expression of cold‐responsive genes) upon subsequent cold exposure, enabling the plant to adapt more efficiently to recurring temperature fluctuations. In this view, cell wall ‘structural memory’ functions not merely as a passive record of environmental history but as an active modulator of future physiological responses, thereby contributing to long‐term environmental adaptation. Further studies to clarify the molecular interface between specific wall modifications and sensor activation will be essential to substantiate this model of cell wall‐based memory.

In trees, for example, temperature is known to influence xylem morphogenesis, including tracheids, which are visually expressed as annual rings. SCW deposition in tracheids occurs just before the cells die and dry out. These rings are commonly used as historical records of growth conditions, such as seasonal temperature or precipitation. For instance, in *Picea abies*, SCW thickening in tracheids positively correlated with summer temperatures, just preserving a visible record of the growing environment (Castagneri *et al*., 2017). Although these anatomical features do not constitute memory in the functional sense, they may have minor effects on future xylem function, such as water transport and mechanical stability, which in turn could influence overall plant performance or ecological interactions, which might be referred to as legacy effects (Sass‐Klaassen *et al*., 2016). However, such legacy effects may reflect passive structural constraints rather than active memory. In this context, we define functional memory as changes that actively modulate subsequent physiological responses, rather than simply persisting as inert structural records.

The concept of structural memory in the cell wall is further supported by evidence from herbaceous species such as Arabidopsis, where even short‐term cold exposure leads to irreversible anatomical changes. In some herbaceous plants, even brief exposure to low temperatures (e.g. 5 d) induces irreversible anatomical changes after the plants return to warmer conditions, as shown in Arabidopsis (Gorsuch *et al*., 2010). While the direct effects of these changes on future stress responses are not yet fully understood, their persistence suggests that the altered anatomical state could predispose tissues to respond differently upon subsequent cold exposure. Such changes could also influence the behavior of plants in response to later cold exposure as a possible component of a structural memory within the cell wall. This ‘structural memory’ in the cell wall, alongside epigenetic mechanisms, may allow plants to balance adaptation and growth in fluctuating environments. However, the structure of polysaccharides and lignin polymers varies not only among species, tissues, and cell types but also across different regions within the cell wall. Therefore, a comprehensive understanding of the cell wall's memory function requires the investigation of both the biochemical building blocks and their spatial organization within the cell wall matrix.

## Memory of cellular spatial information

The cell wall surrounding the cell is not only crucial for information exchange but also plays an active role in storing spatial information that helps regulate cellular organization. In plant cells, the cell wall is a nexus of the PM, and the relationship is well observed in asymmetrically distributed transmembrane proteins, such as transporters, on the PM. In general, such localization patterns reflect cell polarity, which is determined by the spatial information within the tissues or body axes. Interestingly, reduction in cellulose synthesis in *cesa3* mutants led to an abnormal localization pattern of the auxin efflux carrier PIN‐FORMED1 (PIN1), which polarly localizes on the bottom PM at steady state (Feraru *et al*., 2011). Similarly, treatments with cellulose synthase‐specific herbicides, 2,6‐dichlorobenzonitrile (DCB) and isoxaben, resulted in comparable disruptions to the PIN1 polarity (Feraru *et al*., 2011). Additionally, the polar localization of various transmembrane proteins, such as PIN1 and PIN2, ATP Binding Cassette transporters ABCG36 and ABCG37, and borate transporter BOR1, was significantly disrupted following cell wall digestion (Boutté *et al*., 2006; Feraru *et al*., 2011; Łangowski *et al*., 2016). These findings suggest that the polar localization of several transmembrane proteins is generally maintained through the cell wall, and that the polarity or geometry embedded in cells or tissues may be associated with the cell wall.

PM lipids and membrane proteins are widely recognized to exhibit a distinct compartmentalized organization, commonly called nanodomains, for governing cellular processes (Jaillais *et al*., 2024). Given the close physical association of the cell wall with the PM, as well as the interactions with PM‐localized proteins, one of the primary mechanisms by which the cell wall contributes to PM nanodomain formation is through the regulation of lateral diffusion of PM proteins. Martinière *et al*. (2012) demonstrated that the cell wall constrains the lateral diffusion of PM proteins through physical interactions between the extracellular regions of proteins and the cell wall materials. This direct diffusion restriction likely contributes to the compartmentalization and stability of nanodomains. Furthermore, the composition of the cell wall influences nanodomain size and dynamics (McKenna *et al*., 2019; Daněk *et al*., 2020). Altering cell wall composition using the cellulose synthase inhibitor DCB and a PME inhibitor epigallocatechin gallate (EGCG) affects the nanodomain behavior and lateral diffusion of membrane integral proteins such as the flagellin receptor flagellin sensitive 2 (FLS2) and an auxin efflux carrier PIN3 (McKenna *et al*., 2019). Furthermore, the dynamics of nanodomains marked by PM‐associated, intracellular proteins flotillin 2 and hypersensitive induced reaction 1 (HIR1) were similarly affected by the cellulose biosynthesis inhibitor isoxaben, as well as EGCG (Daněk *et al*., 2020). Alternatively, the nanodomain formation could be indirectly controlled by membrane tension or lipid packing associated with the cell wall. Watson *et al*. (2023) demonstrated that the tension or lipid packing on the PM can be increased when the artificial lattice with a transmembrane domain is assembled on part of a mammalian cell surface. Intriguingly, a study with artificial vesicles demonstrated that membrane tension inhibits lipid diffusion (Shendrik *et al*., 2023). These findings imply that, in plants, the clustering of proteins associated with both the PM and the cell wall could alter the physical properties and lipid composition of the PM. These properties could influence the behavior of proteins localized in the region where the PM and cell wall tightly contact. Thus, the cell wall is not merely a structural support for the PM but also regulates the formation and stability of nanodomains by controlling membrane protein localization. This cell wall–PM continuum is essential for the regulation of membrane functions and signal transduction in plant cells (Liu *et al*., 2015; Gorelova *et al*., 2021).

**Fig. 4:**
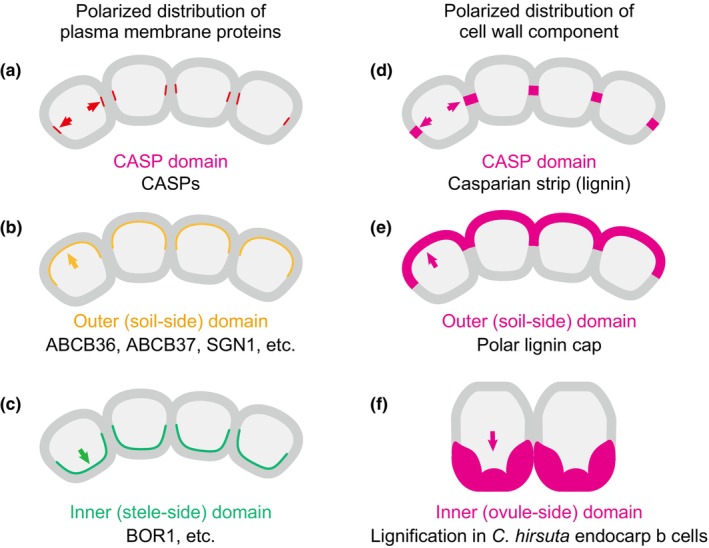
Spatial regulation of the cell wall. (a–c) Membrane‐localized proteins such as Casparian strip membrane domain proteins (CASPs) (a), ATP Binding Cassette (ABC) transporter proteins ABCB36, ABCB37, and SCHENGEN1 (SGN1) (b), and BOR1 (c) are depicted to polarly localize in specific domains of the plasma membrane. (d–f) Lignin, a major secondary cell wall component, is also polarly distributed in the specific cell types. In mature endodermal cells in Arabidopsis, lignin is preferentially polymerized above the CASP domains and forms the Casparian strip that blocks apoplastic solute diffusion (d). In tomato exodermal cells, lignin is polarly deposited in the outer domain of the cell wall and forms the polar lignin cap (e). In *Cardamine hirsuta* endocarp b cells, lignin is polarly deposited to develop explosive pods (f).

Cell wall components, such as lignin, also deposit asymmetrically in cells or tissues, which may reflect the cell polarity (Fig. 4). The Casparian strip, an apoplastic diffusion barrier, is established by localized lignin deposition that is regulated by proteins such as Casparian strip membrane domain proteins (CASPs) and a secreted peroxidase PER64, which are targeted to the PM and the cell wall at the site of strip formation, respectively (Lee *et al*., 2013; Kalmbach *et al*., 2017). Spatially restricted lignification has also been observed in various plant species. For instance, polar deposition of lignin on the outer side is seen in the exodermis of tomato roots (Manzano *et al*., 2024). Similarly, in the outer integument 1 (oi1) cells of the *Arabidopsis thaliana* seed coat, lignin is asymmetrically deposited on the outer side (Hyvärinen *et al*., 2025). Intriguingly, exposure to cold temperature enhances the polar lignification as well as suberization of entire oi1 cells, thereby reducing seed coat permeability. This finding suggests that cold experiences in maternal plants can be recorded as the ‘memory’ in the oi1 cell wall to strengthen the dormancy (Hyvärinen *et al*., 2025). The polar lignification may be partially regulated by polarly localized lignification enzymes in the apoplast, similar to the polar distribution of PER64 in Casparian strip formation. In *Cardamine hirsuta*, polar deposition of lignin in the fruit is essential for explosive seed dispersal, and the lignification enzymes laccases (LACs) and PER66 colocalize with lignin (Pérez‐Antón *et al*., 2022). These findings indicate that localized lignin deposition is regulated by the lignification enzymes polarly localized in the cell wall.

There are several other examples of cell wall components deposited in a polar manner. For example, the polarized tip growth of pollen tubes depends on the spatially regulated remodeling of pectin. PMEs are selectively localized at the tip of the pollen tube through polar secretion, where they regulate pectin status and thus influence cell wall stiffness and elongation (Bosch *et al*., 2005; Wang *et al*., 2013). Besides cell wall‐remodeling enzymes, the secretion of polysaccharides, such as homogalacturonan, is controlled by membrane trafficking systems (Ebine & Ueda, 2015). This process may lead to spatial heterogeneity in the composition of the cell wall, which in turn affects cellular mechanics and signaling. Its physicochemical and mechanical properties appear to persist over relatively long periods, suggesting the possible presence of the memory of the spatial information in the cell wall. A compelling idea is that this long‐lasting cell wall heterogeneity within individual cells may function as cell‐surface landmarks that provide stable cues for nanodomain organization and cell polarity maintenance. As evidence supporting the idea, the polarity indicator protein BASL (breaking of asymmetry in the stomatal lineage), an intracellular protein, was reported to display the polar PM localization even after the protoplast formation; however, the polar localization is unsteady and fluctuating (Chan *et al*., 2020). This indicates that cell polarity can be established independently of the cell wall, whereas the cell wall could function to maintain the established polarity. The intracellular polarity could spatially coordinate the distribution of membrane components, intracellular proteins, cell wall materials, and wall‐remodeling enzymes. This polarity may, in turn, be maintained by the cell wall heterogeneity that acts as the memory of cellular spatial information (see also ‘Receptor‐like kinases monitoring pectin status’ section). This could ensure robust cellular functions even in fluctuating environments. However, we cannot rule out that the cell wall might function merely as a physical scaffold for polarity maintenance in plants, rather than performing higher‐level roles that could be considered ‘memory’. Future studies that visualize distinct cell wall components and demonstrate their direct interactions with polarized or nanodomain‐associated proteins would provide stronger support for the idea that cellular spatial information is encoded as heterogeneity in the cell wall and interpreted by proteins on the PM. The cell wall heterogeneity across tissues may also contribute to the self‐organization of organs, and we discussed that from the aspect of peptide hormone diffusion in the ‘Cell wall components function as apoplastic diffusion constraints?’ section.

## Reading out the memory?

We have outlined how cellular experiences of external/internal environmental changes or geographic traits of cells/tissues can be stored as memories in the cell walls. But how are the memories stored in the cell wall, and if these memories are read out to improve plant fitness, how could they be performed? The latter process could depend on communication within and between the extracellular and intracellular machineries (Fig. 1). The cell wall forms a continuum with the PM through physical links, where extracellular and intracellular events can influence each other (Baluška *et al*., 2003; Liu *et al*., 2015). Plant cells monitor alterations in cell wall architecture in response to environmental/developmental stimuli, which is called CWI control. The well‐known mechanism to maintain CWI comprises cell wall sensors localized on the PM and their downstream signaling components in the cells. Recent findings on the CWI mechanism have been thoroughly reviewed (Ringli, 2010; Baez *et al*., 2022; Wolf, 2022; Oelmüller *et al*., 2023). Here, we highlight the key features and present a concept of how plants can use information stored in the cell wall.

### Receptor‐like kinases monitoring pectin status

**Fig. 5:**
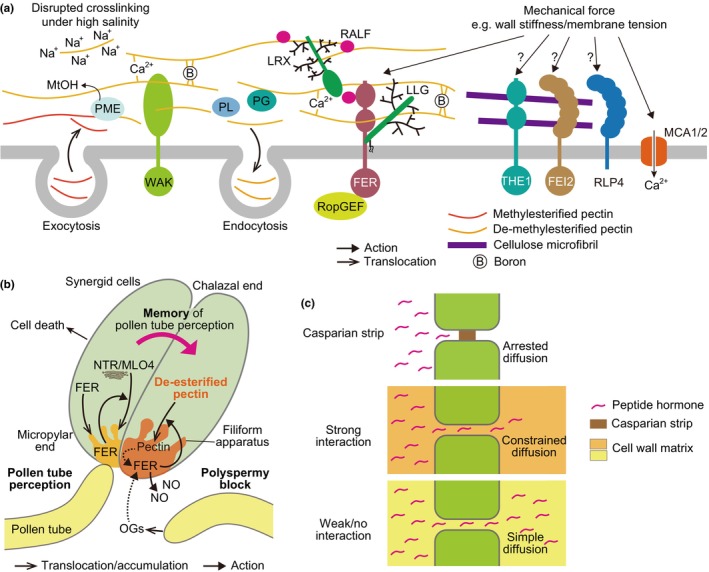
Reading out cell wall memories. (a) Recognition machineries of cell wall status. Receptor‐like kinases wall‐associated kinase (WAK) and FERONIA (FER) recognize pectins, particularly those in de‐methylestelized state. Newly synthesized methylesterified pectin is transported into the cell wall by exocytosis. Pectin methylesterase (PME) removes methyl ester groups from pectins, producing methanol (MtOH) as a byproduct. While de‐methylesterified pectins are cross‐linked with calcium ions or boron, high salinity conditions disrupt the calcium cross‐linking. Pectins are also digested by polygalacturonase (PG) or pectate lyase (PL), and the fragmented pectins are removed from the cell wall by endocytosis. WAK and FER can monitor these changes in the pectin status of the cell wall. Glycoproteins, leucine‐rich repeat extensin proteins (LRXs), LORELEI‐LIKE glycosylphosphatidylinositol‐anchored proteins (LLGs), and secreted peptide‐ligands rapid alkalinization factors (RALFs) may help FER to recognize the pectin status. THESEUS1 (THE1) and FEI2 may bind cellulose to mediate cell wall damage response. Receptor‐like protein 4 (RLP4) binds to unknown components of the cell wall. They may monitor the mechanical status of the cell wall. Mechanical force can influence the membrane tension through the cell wall, and the mechanosensitive calcium ion channels mid1‐complementing activity 1 and 2 (MCA1/2) monitor it. Arrows indicate action or translocation, which are specified in the panel. (b) A cell wall memory in Arabidopsis fertilization. FER regulates both pollen tube perception and polyspermy block. While one of two synergid cells accepts a pollen tube for fertilization and then dies, the other increases de‐methylesterified (de‐esterified) pectin at the filiform apparatus, a specialized structure at the micropylar end, to prevent further pollen tube entries (storing a memory in the cell wall). FER is localized at the filiform apparatus by polarized trafficking before and after pollination and regulates the pectin accumulation. Upon further pollen tube approaches, where oligogalacturonides (OGs), fragmented pectin, could be released from pollen tubes, FER activates production of nitric oxide (NO) at the filiform apparatus, thereby blocking additional pollen tube avoidance. The accumulated pectin at the filiform apparatus could enhance FER signaling through its immobilization. In pollen tube perception, FER regulates the trafficking of NORTIA/midrew locus O 4 (NTR/MLO4) from the Golgi to the plasma membrane. Arrows indicate action or translocation/accumulation, which are specified in the panel. Arrows with dashed lines indicate plausible actions that require further investigation. (c) A model of apoplastic diffusion constraints. The Casparian strip seals the apoplastic space between the cells with lignin and suberin, which are hydrophobic compounds, thereby blocking apoplastic diffusion of peptide hormones (top panel). Even if the Casparian strip is absent, apoplastic diffusion could be influenced by the cell wall matrix. When peptide hormones strongly interact with the matrix, their diffusion can be diminished (middle panel). When peptide hormones weakly or rarely interact with the cell wall matrix, their diffusion is almost dependent on their molecular size, referred to as simple diffusion (bottom panel).

Wall‐associated kinases (WAKs) and *Catharanthus roseus* receptor‐like kinase 1‐like (CrRLK1‐like) kinases are well‐characterized receptor‐like kinases (RLKs) that act as CWI sensors. WAKs have crucial roles in cell elongation, cell differentiation, and plant–pathogen interactions (Oelmüller *et al*., 2023). The extracellular domain of Arabidopsis WAK1 binds calcium cross‐linked pectin or oligogalacturonides (OGs), which are in the de‐methylesterified state, and are activated upon binding (Decreux & Messiaen, 2005; Decreux *et al*., 2006; Brutus *et al*., 2010). This suggests that WAK1 binds to structural pectins in the cell wall to monitor the status or amount of pectin during development and environmental responses. The following alterations in the cell wall may modulate WAKs‐mediated responses: the deposition of synthesized methylesterified pectin, endocytosis of pectin, de‐esterification of pectin by PMEs in the cell wall, and changes in the apoplastic ionic environment (Baluška *et al*., 2002; Decreux & Messiaen, 2005) (Fig. 5a). However, we have to be aware that a recent report demonstrated that WAKs are not required for the OG‐induced immune signaling and immunity by using quintuple mutants lacking *WAK1* to *WAK5* (Herold *et al*., 2025). Their binding to OGs and pectin and the involvement in growth and development need to be further investigated.

FERONIA (FER), the most studied CrRLK1‐like kinase, was initially identified as a crucial factor for female fertility, but an increasing number of reports have shown its highly diverse roles in various cellular processes, including hormonal signaling, defense responses, and mechano‐sensing (Deslauriers & Larsen, 2010; Duan *et al*., 2010; Shih *et al*., 2014; Cheung, 2024). The extracellular domain of FER binds highly de‐methylesterified polygalacturonic acid, which is the backbone of structural pectin and longer than OGs (Feng *et al*., 2018). FER also recognizes secreted peptide‐ligands, rapid alkalinization factors (RALFs), together with the cell wall‐localized leucine‐rich repeat extensin proteins (LRXs) or with the PM‐bound co‐receptor LORELEI‐LIKE glycosylphosphatidylinositol‐anchored proteins (LLGs) to activate the RALF signaling cascades (Zhao *et al*., 2018; Dünser *et al*., 2019; Xiao *et al*., 2019) (Fig. 5a). FER accumulates in dynamic foci, that is, nanodomains, on the PM (Gronnier *et al*., 2022; Liu *et al*., 2024). Recent findings demonstrated direct binding of RALFs to pectin. RALF1 binds to short pectin fragments produced by environmental changes or pathogen attacks through the electrostatic interaction between negatively charged RALF1 and positively charged, de‐methylesterified pectin (Liu *et al*., 2024; Rößling *et al*., 2024). They form condensates in the cell wall, facilitating FER nanodomain formation with LLG1 to control cellular processes. Intriguingly, RALFs compact de‐methylesterified homogalacturonan (HG), the major component of structural pectin, by acting together with cell wall‐localized LRXs in pollen tubes and root hairs. The RALF‐LRX complexes are widely distributed in their cell walls, where the complex may have roles in cell wall assembly, which is not related to typical FER signaling (Moussu *et al*., 2023; Schoenaers *et al*., 2024). These traits may facilitate FER function in monitoring pectin status throughout the cell wall.

The function of FER in fertilization might shed light on how cellular ‘memories’ are formed and utilized in cell walls (Fig. 5b). FER is broadly expressed, including the synergid cells that compose the female gametophyte (Escobar‐Restrepo *et al*., 2007; Duan *et al*., 2020). The synergid cell is a polarized cell exhibiting an asymmetric cell wall. The cell wall is absent or discontinuous at the chalazal pole, while at the micropylar end, its extension is invaginated into the cell, forming a unique structure called the filiform apparatus (Kasahara *et al*., 2005). FER is accumulated at the filiform apparatus, regulating pollen tube perception and polyspermy blocking (Duan *et al*., 2014; Li *et al*., 2015; Mizuta *et al*., 2024) (Fig. 5b). The filiform apparatus includes cellulose, hemicellulose, pectin, callose, and proteins (Drews & Koltunow, 2011). In Arabidopsis, de‐methylesterified pectin is abundant at the filiform apparatus, and pollination increases its level in pistils containing ovules in a FER‐dependent manner (Duan *et al*., 2020) (Fig. 5b). A similar observation was reported in *Hyacinthus orientalis*: an increased level of Ca^2+^‐associated HG at the filiform apparatus after pollination (Niedojadło *et al*., 2015). Multiple pollen tubes were frequently observed close to the ovules in the *pme33/44* mutants and *PME11* overexpressor, while the frequency for wild‐type ovules was low (Duan *et al*., 2020), implying that the methylesterification status at the filiform apparatus could influence the signal transduction to block the attractance of further pollen tubes after pollination. An attractive explanation is that the accumulation of de‐methylesterified pectin could strengthen FER signaling by immobilizing FER at the filiform apparatus. This immobilization could occur because FER has a high affinity for highly de‐methylesterified pectin, and its PM localization depends on the association with pectin through its extracellular malectin‐binding domain (Feng *et al*., 2018; Lin *et al*., 2022). Upon further pollen tube approaches, leading to the release of pectin fragments, FER signaling triggers nitric oxide (NO) production at the filiform apparatus (Duan *et al*., 2020) (Fig. 5b). NO can modify the pollen tube attractant LURE1 peptide and change its activity or trafficking, thereby preventing polyspermy. These findings imply that the ‘memory’ of pollination is ‘stored’ as the pectin status in the filiform apparatus cell wall. As a downstream ‘readout’, this altered status could enhance FER signaling while also stiffening the cell wall, thereby blocking additional pollen tube entries. This hypothesis will be confirmed by investigating whether the genetic modulation of PMEs activity at the filiform apparatus could affect the NO accumulation. In a similar context to synergid cells, FER is tightly associated with the pavement cell wall, where de‐methylesterified pectin asymmetrically accumulates, and the interaction between FER and highly de‐methylesterified pectin regulates the morphogenesis through the ROP6 pathway (Lin *et al*., 2022). Mechanical stress also induces ROP6 activation via FER that associates with de‐methylesterified pectin (Tang *et al*., 2022). Thus, the pectin status in the cell wall of these cells might act as ‘memory’ for fertilization or mechanical stress, which is monitored by FER for further responses.

FER regulates not only the accumulation of de‐methylesterified pectin but also the trafficking of NORTIA/MLO4 (mildew resistance locus O 4), a downstream factor of FER signaling, from the Golgi to the filiform apparatus during pollen tube perception (Ju *et al*., 2021) (Fig. 5b), suggesting the role of FER in local, polarized accumulation/trafficking of cell wall components and proteins. The cell polarity might be maintained by feedback‐loop mechanisms – asymmetric accumulation/targeting of cell wall components and/or sensing them – regulated by CWI signaling. Further investigation is needed to determine if the asymmetric distribution of cell wall components, such as pectin, leads to a similar accumulation pattern of PM proteins, such as CWI sensors.

### Potential sensors for cell wall status, including mechanical force

The sensing mechanisms for other cell wall components are less well‐known compared to those for pectin. The cellulose biosynthesis inhibitor isoxaben induces the response to cell wall damage (CWD), and several PM‐localized RLKs, for example, CrRLK1‐like THESEUS1 (THE1) and leucine‐rich repeat RLK FEI2, have been shown to contribute to the responses (Hématy *et al*., 2007; Basu *et al*., 2016; Van der Does *et al*., 2017; Chaudhary *et al*., 2020; Bacete *et al*., 2022). However, it is unknown whether those RLKs recognize changes in the cellulose itself. Engelsdorf *et al*. (2018) have reported that a key element in CWD responses is the sensing mechanism of mechanical and hypo‐osmotic stresses. This finding suggests that plants monitor the stiffness of the cell wall as it depends on cell wall composition, besides recognizing cell wall components directly through receptors (Fig. 5a). To give another example, the recently discovered receptor‐like protein RLP4, which is localized at the cell edges through interaction with some cell wall components, monitors the mechanical status of the cell wall (Elliott *et al*., 2024). Given that the tension of the PM can be controlled by the cell wall, as discussed in the previous section, the ion channels MCA1/2 (mid1‐complementing activity 1 and 2), which monitor membrane tension (Yoshimura *et al*., 2021), may also constitute one of the mechanisms that detect the mechanical state of the cell wall (Fig. 5a). Looking from a different angle, immunity triggered by damage‐associated molecular pattern (DAMP) provides us with hints on how plants monitor the cell wall status. DAMPs are released when a pathogen disrupts the cell wall or PM (Hou *et al*., 2019). Specifically, DAMPs derived from plant cell walls, such as cutin monomers from the cuticle, OGs from pectin, cellooligomers (cellobiose, cellotriose, cellotetraose, and short‐chain glucose polymers) from cellulose, xyloglucan oligosaccharides from hemicellulose, and methanol from pectin (produced by PME), are reported to activate defense responses through cellular signaling in plants (Hou *et al*., 2019). Plants may use identical or similar systems for monitoring the cell wall status in development or other environmental responses, as they do for defense responses. This is because these DAMPs can be produced by normal plant activities, and defense signaling is closely linked with growth‐related hormone signaling, a phenomenon referred to as defense–growth tradeoffs (Huot *et al*., 2014). Supporting this, the IGP/CORK (impaired in glycan perception/cellooligomer‐receptor kinase) proteins are characterized in both CWI and immune responses triggered by oligosaccharides derived from plant cell wall β‐glucans, such as mixed‐linked glucans (MLG43 and MLG34) or cellulose (cellotriose, cellotetraose, and cellopentaose) (Tseng *et al*., 2022; Martín‐Dacal *et al*., 2023). IGP/CORKs are receptor‐like kinases including leucine‐rich and malectin domains (LRR‐MAL‐RLKs), and the extracellular domain of IGP1/CORK1 and IGP3 directly bound to cellotriose but not to MLG43, suggesting that IGP/CORKs could recognize those oligosaccharides as sole or together with other recognition proteins (Martín‐Dacal *et al*., 2023). The receptors for these DAMPs, except for WAKs and FER against OGs and IGP/CORKs for cellulose, still need to be identified.

### Role of RLKs and calcium signaling during cold acclimation

Leucine‐rich receptor‐like kinases (LRR‐RLKs) play pivotal roles in cold response in plants. In Arabidopsis, CRLK1, a calcium/calmodulin‐induced LRR‐RLK, is localized on the PM and involved in cold tolerance (Yang *et al*., 2010; Furuya *et al*., 2013). GsLRPK from *Glycine soja*, phloem intercalated with xylem‐like 1 (PXL1), and kinase on the inside (KOIN) from Arabidopsis, and cold tolerance LRR‐RLK1 (CTLK1) from Medicago are also localized on the PM and control cold tolerance (Yang *et al*., 2014; Jung *et al*., 2015; Geng *et al*., 2021; Zhang *et al*., 2025). Some of these LRR‐RLKs or their relatives could likely recognize cell wall components such as l‐arabinose in pectin during cold acclimation and deacclimation (see the ‘Memory of temperature changes’ section) because WAKs and FER also belong to LRR‐RLKs (Shiu & Bleecker, 2001). Another type of RLKs also controls cold tolerance; L‐type lectin‐RLKs (LecRLKs) can bind hydrophobic ligands like monosaccharides or polypeptides, and heterogenous overexpression of a PM‐bound LecRLK, which is induced by various stresses including cold stress in a moss, *Pohlia nutans*, leads to enhanced cold tolerance in Arabidopsis (Liu *et al*., 2017, 2023). These findings suggest RLKs control cold tolerance by monitoring extracellular environments, possibly cell wall components.

Calcium signaling functions with RLKs or may act as an indirect mechanism for perceiving structural alterations triggered by cold acclimation. One well‐characterized aspect of cold acclimation involves a transient elevation in cytosolic Ca^2+^ concentration in response to temperature drops, which acts as a primary trigger for the cold‐responsive transcriptional cascade. This cytosolic Ca^2+^ is sensed by calmodulin, which binds to calmodulin‐binding transcription activator 3 (CAMTA3) and CAMTA5, leading to the induction of CBF/DREB1 (C‐repeat binding factor/dehydration‐responsive element binding 1) transcription factors, which are master regulators of cold acclimation (Fowler & Thomashow, 2002; Doherty *et al*., 2009; Kidokoro *et al*., 2017). CRLK1, the cold stress‐related RLK, also interacts with calmodulin and phosphorylates MEKK1 (mitogen‐activated protein kinase kinase kinase 1), while cold stress induces the phosphorylation dependent on Ca^2+^, enforcing the relationship between Ca^2+^ signaling and RLK in cold stress response (Yang *et al*., 2010; Furuya *et al*., 2013).

Much of this cytosolic Ca^2+^ is thought to originate from the apoplast. The cell wall, through HG and AGPs, can bind and retain Ca^2+^ (Lopez‐Hernandez *et al*., 2020). Cold‐induced structural remodeling of the cell wall, including de‐methylesterification of HG and glycan modification of AGPs, may modulate the amplitude and temporal characteristics of cold‐induced Ca^2+^ signals by altering Ca^2+^ availability. Supporting this idea, cold‐induced high activity of PME, which mediates HG de‐methylesterification, has been reported to persist even during the deacclimation phase (Solecka *et al*., 2008), suggesting that the cell wall can maintain a primed state for Ca^2+^ signaling. Therefore, a structural memory of prior temperature changes may be encoded through Ca^2+^ signaling plasticity mediated by cell wall remodeling, making it accessible during future stress responses. This proposed role offers mechanistic insight into how structural memory in the cell wall can influence intracellular cold signaling through both biochemical and biophysical pathways.

### Downstream signaling of CWI sensors

The alteration of the cell wall status can trigger various cellular events on different time scales. The cytosolic parts of CrRLK1‐like FER and THE1 consist of serine/threonine kinases that phosphorylate target proteins such as guanine nucleotide‐exchange factors (GEFs) or ROPs (Baez *et al*., 2022). Phosphorylation of RopGEFs activates ROPs, leading to downstream events including reactive oxygen species production and microtubule rearrangement (Tang *et al*., 2022). The FER‐mediated phosphorylation cascade also inhibits PM H^+^‐ATPase activity (Haruta *et al*., 2014). As another aspect of FER‐mediated signaling, FER controls not only transcription but also alternative splicing (Wang *et al*., 2020; Cheung, 2024). Hormonal signaling of ethylene, jasmonic acid, salicylic acid, abscisic acid, and auxin shows crosstalk with FER signaling. Other CWI sensors are involved in some of the above or other specific events. These mechanisms may explain how cell wall memories are stored and used to control various processes in plants during the various periods described above.

### Cell wall components function as apoplastic diffusion constraints?

The interaction of RALF peptide hormones with pectin suggests that their diffusion is constrained by cell wall composition. Secreted peptides are generally thought to move intercellularly through the apoplast via simple diffusion (Matsubayashi, 2011, 2014, 2018). However, diffusion in complex matrices such as apoplastic space, including the plant cell wall, is likely influenced by matrix structure, solute properties, and their interactions (Fig. 5c). An attractive hypothesis is that local accumulation of cell wall components in a cell or tissue, that is, cell wall heterogeneity, modulates the diffusion rate or flow of peptide hormones, as a consequence of geometric ‘memories’ in organ development and growth. Such effects could determine the distribution of peptide hormones in tissues but have rarely been considered, except in the context of the Casparian strip, a known apoplastic diffusion barrier composed of lignin and suberin (Lalun & Butenko, 2025). Proline hydroxylation is a posttranslational modification found in many plant peptide hormones, including plant peptides containing sulfated tyrosine 1 (PSY1), tracheary element differentiation inhibitory factor (TDIF), CLAVATA 3 (CLV3), root meristem growth factor 1 (RGF1), and Casparian strip integrity factors (CIFs) (Matsubayashi, 2011; Nakayama *et al*., 2017). Some, such as CLV3, which controls the stem cell homeostasis in the shoot apical meristem, are further modified with an *O*‐linked l‐arabinose chain. While these modifications are important for biological functions like receptor binding, they may also influence diffusion by promoting hydrogen bonding with cell wall components through hydroxyl groups in hydroxylated proline residues or sugar chains. Indeed, the distribution patterns of some cell wall components, such as cellulose and heteromannans, are uneven in the shoot apical meristem (Yang *et al*., 2016).

Sulfation of tyrosine is often detected at the second residue of secreted peptide hormones like PSY1, RGFs, and CIFs (Matsubayashi, 2018), and that might affect the distribution or diffusion rate of CIFs in tissues. CIFs, including TWS1 (twisted seed1), regulate extracellular barrier formation (Fujita, 2021). CIF1/2, which are expressed, processed, and sulfated in root stele cells, are diffused in the apoplast and recognized by their receptors GASSHO1/SCHENGEN3 (GSO1/SNG3) and GSO2 at endodermal cells to form the Casparian strip. In immature seeds, unprocessed TWS1, which is expressed and sulfated in the embryo, passes through the embryonic cuticle and is then processed by a secreted peptidase expressed in the endosperm. The mature peptide re‐crosses the cuticle to activate GSO1/2 for the cuticle formation. The sulfation of TWS1 increases the accuracy of its proteolytic processing for maturation, suggesting that the sulfation may facilitate the bioactivity of TWS1 (Royek *et al*., 2022). Sulfation is also known to be important for the binding activity of peptides to their receptors, and it is indispensable for the role of RGF1 in maintaining root meristem size (Song *et al*., 2016). Like RGF1, nonsulfated CIFs greatly reduce their affinities against GSO1/2 *in vitro*; however, they still have residual bioactivities in Casparian strip formation in roots or embryonic cuticle formation (Doll *et al*., 2020; Okuda *et al*., 2020). Such a discrepancy has not been fully explained. Tyrosine sulfation can change molecular properties, such as surface charge and structure, and these effects could be enhanced as peptides rather than larger precursor proteins, which might impact their diffusion rate in tissues through interactions with cell wall components. Future research focusing on these topics, like the visualization of peptide hormones in tissues, will give new insights into cell–cell communication in self‐organized development and environmental responses.

## Conclusion

Growing evidence has supported the idea that, in plants, the cell wall provides information storage that is apart from the epigenetic context. The hysteresis of the cell wall, which integrates information up to the present, enables plants to adapt to fluctuating environments by affecting morphogenesis (e.g. cold acclimation). Well‐known examples of ‘memory’ in biology include the memory implemented by the central nervous system and the immune memory in animals, as well as epigenetic memory in all forms of life. However, a broader survey across the kingdoms of life suggests not a few instances where memory is retained in the extracellular space. For instance, in budding yeast, budding avoids sites called budding scars left on the cell wall, as if the mark prevents subsequent budding at the same site. Similarly, animal cells regulate organogenesis and cellular function through the construction of the extracellular matrix. These examples imply that organisms do not rely solely on DNA or chromatin modifications but also utilize extracellular space as a site of memory. Further research in this context will reveal that the cell wall is not merely a static or dynamic structure, but also functions as a multilayered memory system. Progress in this direction will not only lead to new developments in the study of cell wall functions but also deepen our understanding of how plants form memories, as well as the physiological, ecological, and evolutionary implications of these memories.

## Competing interests

None declared.

## Author contributions

HS, AY, MA and DT developed the ideas presented here, drafted, and revised the manuscript collaboratively.

## Disclaimer

The New Phytologist Foundation remains neutral with regard to jurisdictional claims in maps and in any institutional affiliations.
